# Dose perturbation in the radiotherapy of breast cancer patients implanted with the Magna‐Site: a Monte Carlo study

**DOI:** 10.1120/jacmp.v12i2.3295

**Published:** 2011-01-19

**Authors:** Christos Chatzigiannis, Georgia Lymperopoulou, Panagiotis Sandilos, Constantinos Dardoufas, Emmanouil Yakoumakis, Evaggelos Georgiou, Pantelis Karaiskos

**Affiliations:** ^1^ Department of Radiology Areteion University Hospital Athens Greece; ^2^ Department of Medical Physics Medical School, University of Athens Greece

**Keywords:** radiotherapy, magna site, breast, Monte Carlo

## Abstract

External beam radiation therapy (RT) is often offered to breast cancer patients after surgical mastectomy followed by breast reconstruction with silicone implants. In some cases, the RT is administered while the patient is still implanted with a temporary tissue expander including a high‐density metallic port, which is expected to affect the planned dose distribution.

This work uses Monte Carlo (MC) simulation in order to evaluate the aforementioned effect when the McGhan Style 133 Tissue Expander with the Magna‐Site injection port is used. Simulations have been performed on a patient model built using the actual CT images of the patient for two irradiation schemes, involving two tangential photon beams of 6 MV and 18 MV respectively. MC results show that the presence of the Magna‐Site within the two irradiation fields leads to an overall reduction of absorbed dose for points lying in the shadow of the metallic port (relative to each of the opposing beams). The relative reduction compared to dose results without the expander in place ranges from 7% to 13% for the 6 MV beam and is around 6% for the 18 MV photon beam. However, in the close vicinity of the metallic port, increased absorbed doses are observed, due to the increase of secondary electrons emerging from the metallic part of the insert.

PACS numbers: 87.53.Bn, 87.55.K‐, 29.20.‐c, 87.56.bd, 75.50.‐y

## I. INTRODUCTION

Reconstruction of the breast is the usual choice of breast cancer patients that undergo surgical mastectomy. The procedure is typically completed within two surgical interventions. The first stage involves the subcutaneous or submuscular implanting of a tissue expander at the site of the mastectomy, which is then gradually filled with dilute saline solution injections through an injection port to the desired volume. Finally, at a second surgical stage, the tissue expander is removed and replaced by a permanent implant.

High‐risk breast cancer patients, as well as intermediate risk patients, are often referred for adjuvant radiation therapy, since this is shown to increase long‐term survival, on the condition that appropriate radiotherapy techniques are being used.^(1)^ Many of these patients undergo RT before the second phase of breast reconstruction with the expander in place.

Since many of the clinically used tissue expanders contain high‐Z metallic ports with complicated apertures which usually lie within the treatment fields, questions have arisen about the dosimetric effect on the planned dose distribution of the RT scheme. One of the major concerns is whether the high‐density material perturbs the photon RT fields in such a way that small areas in the chest wall region can undergo a dose reduction, appearing in the chest wall region, with a potential impact on the RT outcome.

The McGhan Style 133 Tissue Expander (INAMED Aesthetics (formerly McGhan Medical Corporation), Irvine, CA) is one of those expanders intended for temporary implantation. One of its components is the Magna‐Site injection port which contains a high‐density rare‐earth permanent magnet and is intended to be a convenient injection system.^(2)^ The most common rare‐earth magnets are Neodymium and Samarium‐Cobalt magnets with density that varies from approximately $7.4\;{g/\text{cm}}^{3}$ for Neodymium to $8.4\;{g/\text{cm}}^{3}$ for Samarium‐Cobalt magnets.

A limited number of investigations have been performed so far on the specific effect on the radiotherapy treatment due to the metallic port that is present in some of the clinically‐used tissue expanders. Moni et al.,^(3)^ used both film and thermoluminescent dosimeters (TLD) in their *ex vivo* study measuring the dosimetric changes around the metallic port in the McGhan elastic silicone rubber tissue expander, irradiated by a single 6 MV photon beam. Results showed no hot spots, but a decrease in measured dose only throughout the direct shadow of the metallic port.

The effect of the McGhan Style 133 Tissue Expander has been investigated by Thompson and Morgan^(4)^ using diode dosimetry in a water phantom. The underdosage of regions surrounding the magnet was up to ~ 30%, for a single 6 MV photon beam, while it was estimated to be of the order of ~ 10% in a clinical case, when the irradiation was performed with two tangential beams. Damast et al.^(2)^ have studied the effect of the same tissue expander using film dosimetry of a single beam on a water phantom, and TLD and film *in vivo* measurements of patients’ exit dose. They have observed that a small area can undergo a dose reduction of as much as 22% for a single 6 MV photon beam, and 16% for a 15 MV beam, and recommend the use of 15MV photons with compensating bolus for RT treatment.

In this study, we evaluate the dose perturbation of the McGhan Style 133 Tissue Expander in a radiotherapy treatment of the breast, with the aid of Monte Carlo simulation of an actual breast patient implanted with the expander. The first step was to fully simulate the two photon beams (6 MV and 18 MV) generated by the hospital's linear accelerator (linac), using the OMEGA package and the EGSnrc Monte Carlo code. Results have been crosschecked by performing experimental measurements with an ionization chamber in a water phantom. Then, an actual RT treatment of a breast patient has been simulated, using the CT slices of the patient with the tissue expander in place, and comparison has been performed between the dose distributions obtained with and without the presence of the expander. Results are presented in terms of relative dose profiles along selected axes in the central axial plane of the patient's CT, for the two different energy modes of the linac.

## II. MATERIALS AND METHODS

### A. Photon beam modeling

#### A.1 Monte Carlo simulations of linac's head

The OMEGA/BEAMnrc^(^ ^5^ ^,^ ^6^ ^)^ package by the Canadian National Research Council was installed in order to simulate the linear accelerator operating in our radiotherapy department. The package uses the EGSnrc Monte Carlo code and basically consists of the BEAMnrc and DOSXYZnrc user codes^(7)^ to run the simulation.

The linac in our department is a dual‐photon Siemens Primus‐II (Siemens AG, Munich, Germany) working in two nominal modes for photons of 6 MV and 18 MV. It includes a multileaf collimator of 29 double focused opposed leaf pairs. The 27 inner leaves project a shadow width of 1 cm at the isocenter plane, while the two outer leaves have a shadow width of 6.5 cm.

The linac's head was simulated in detail with the BEAMnrc user code, using the following components : a gold target in a stainless steel container, a cylindrical aluminum absorber (for 18 MV mode only), a tungsten primary collimator defining a conical aperture to place the flattening filters inside, the dose chamber consisting of three aluminum layers that define two air volumes, the mirror as common glass with an aluminum layer, a pair of focused tungsten jaws, and 29 tungsten double‐focused leaves defining the MLC collimator.

A Pentium4 2.8 Ghz computer (Intel Corporation, Santa Clara, CA) running under Linux was used to run the EGSnrc Monte Carlo simulation. A number of electron histories ranging between 5×10 ^6^ and 15×10 ^6^, were used according to the field and energy mode of the simulation. The phase space files produced by the code varied from 1 to 10 GB, while the simulation total running time varied from 36 to 120 hrs. The lower energy cutoff's for particle transport were set to AE=700 KeV for electrons and AP=10 KeV for photons.^(^ ^8^ ^,^ ^9^ ^)^ The selective Bremsstrahlung splitting (SBS) was applied as a variance reduction technique with the parameters $N_{\text{min}}=20$ and $N_{\text{max}}=200$.^(^ ^8^ ^,^ ^10^ ^)^ Neither the Russian roulette or any other electron range rejection technique was used.

The phase space files produced from the BEAMnrc code were then used repeatedly, as “source” for the DOSXYZnrc EGSnrc Monte Carlo code. A water tank was simulated consisting of 100×100×100 voxels. The size of the voxels varied from $0.5\times 0.5\times 0.5\;{\text{cm}}^{3}$ to $0.2\times 0.2\times 0.2\;{\text{cm}}^{3}$ near the penumbra of the radiation field and the buildup region, in order to maintain a good resolution of the dose, near regions of steep dose gradients. Depth‐dose curves were calculated for the $10\times 10\;{\text{cm}}^{2}$ field and were normalized at the depth of maximum dose. The in‐plane and cross‐plane profiles for a $10\times 10\;{\text{cm}}^{2}$ and a $20\times 20\;{\text{cm}}^{2}$ radiation field were calculated and normalized at the depth of 10 cm to minimize any effect of contaminant electrons to the calculated dose.^(11)^ The total number of photon histories varied between 5×10 ^8^ and 6×10 ^8^ in order to keep the dose error less than 1%.

The experimental measurements were performed using a 0.125 cm^3^ ion chamber mounted on an automated Blue Scanditronix Wellhofer phantom (IBA Dosimetry GmbH, Schuarzenbruck, Germany). The results were analyzed with the OmniPro Accept Software (IBA Dosimetry GmbH, Schuarzenbruck, Germany).

With the aid of the automated phantom, percent depth‐dose curves have been measured for the 6 MV and 18 MV photon beams, with $10\times 10\;{\text{cm}}^{2}$ field size at a focus‐to‐surface distance (FSD) of 100 cm. In addition, dose profile measurements were performed for $10\times 10\;{\text{cm}}^{2}$ and $20\times 20\;{\text{cm}}^{2}$ fields at FSD=100 cm, at the depth of 10 cm. The total uncertainty of experimental measurements was below 3%.

### B. Monte Carlo simulation of breast patient

The whole RT scheme of a breast patient with a McGhan Style 133 Tissue Expander in place and undergoing radical radiotherapy was simulated in this work. For planning purposes, the patient was scanned on an extended CT‐scale (up to 6000 HU) Siemens SOMOTOM Plus 4 CT scanner (Siemens AG, Munich, Germany) with 3 mm slice thickness.

CT slices in DICOM3 format were processed to create a CT‐based geometry readable from the BEAMnrc Monte Carlo program. For this purpose, the program CTCREATE from the OMEGA package was used. Two CT based geometries were constructed with voxel size $0.2\times 0.2\times 0.3\;{\text{cm}}^{3}$. The first was built to be homogenous (i.e., disregarding the presence of the tissue expander), assigning the density of water $\left(1{g/\text{cm}}^{3}\right)$ to every pixel independently of its HU number. The second was constructed taking into account the inhmogeneities inside the breast, including the aperture of the McGhan Tissue Expander. However, the presence of metallic prostheses resulted in incomplete CT number (HU) data or gaps due to the strong attenuation of the low‐energy X‐rays used in CT that prevent photons from reaching the detector. Thus, the so‐called “hollow” projections are seen that produce lower CT numbers of the implants as well as of structures close to the implants (in the case of the investigated geometry on the breast tissue close to the implant).^(^ ^12^ ^,^ ^13^ ^)^ In this work to reduce this effect, all voxels with HU < 1000 were assigned as water, voxels with 1000< HU < 3500 as titanium, and voxels with HU ≥ 3500 were assigned as neodymium iron boron magnet. In this way the geometry of the tissue expander was reconstructed with an accuracy in the order of 1–2 voxel sizes used for MC simulations $\left(0.2\times 0.2\times 0.3\;{\text{cm}}^{3}\right)$. For MC simulation of the inhomogeneous geometry, a physical density of 4.54 and $7.4\;{g/\text{cm}}^{3}$ was used for the titanium material and the neodymium iron boron magnet, respectively.

The whole RT treatment was simulated with the Monte Carlo OMEGA package, accurately modeling the patient's treatment plan setup, which involved two opposite tangential fields of size $11\times 15.8\;{\text{cm}}^{2}$. The number of histories used for the two beams were normalized according to the MU from each beam. The total number of histories simulated varied from 4×10 ^8^ to 12×10 ^8^ in order to keep the statistical error less than 3%. The same total number of histories was used for the two CT‐based geometries. Monte Carlo results in the homogenous geometry were compared to corresponding TPS calculations without inhomogeneity correction. The output of both RT simulations were used to extract profiles of the radiated breast in various directions in order to estimate the dose perturbation caused due to the presence of the tissue expander.

## III. RESULTS & DISCUSSION

### A. Photon beam modeling

MC calculated photon beam distributions in a water phantom, produced by different characteristics of the electron beam hitting the target inside the linac's head (energy distribution, mean energy, radius and radial distribution of electron beam), were compared to the experimentally measured distributions of the photon beams (nominal energies of 6 MV and 18 MV). The fine‐tuning of the electron beam characteristics has been based to dose profile comparisons in order to determine the beam radius (Verhaegen and Seuntjens,^(14)^ Lin et al.^(10)^), and to depth dose curves in order to determine the energy of the electron beam. Calculations of the depth dose curves showed the insensitivity of the depth dose profiles to the initial electron beam characteristics.^(^ ^15^ ^,^ ^16^ ^)^

After fine‐tuning the initial electron beam characteristics, the parameters that more accurately reproduce the experimental measurements were determined to be a uniform energy distribution of 6.5 MeV for the 6 MV modality, with uniform radial intensity and 2 mm radius. Concerning the18 MV modality, the electron beam was again chosen to be uniform with a 2 mm radius and mean energy 13.5 MeV.

**Figure 1:**
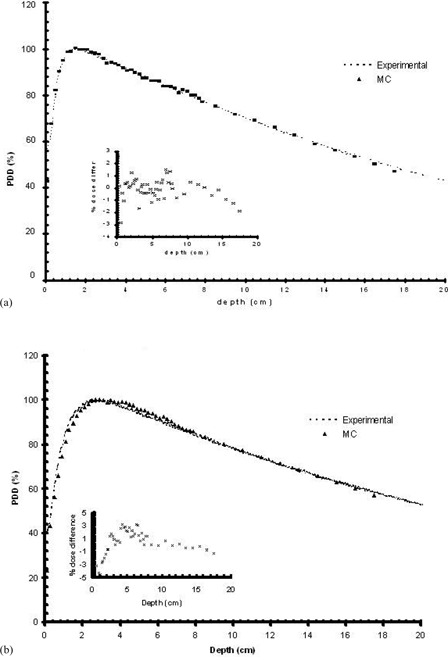
Percentage depth‐dose curve for a 10 by 10 cm^2^ photon field, measured experimentally and as calculated by MC for: (a) the 6 MV mode simulating a 2 mm radius electron beam hitting the target with a uniform energy of 6.5 MeV and uniform electron intensity (upper) and (b) the 18 MV mode simulating a 2 mm radius electron beam hitting the target with a uniform energy of 13.5 MeV and uniform electron intensity (lower). The percentage dose difference is also presented in the figure inset.

Figure 1 presents the depth‐dose curves of the photon beams used in this work for the MC simulations of the breast patient, together with the corresponding experimental results. The close agreement of MC and experimental results for the nominal energies of 6 MV and 18 MV can be observed in (Figs. 1a)and (1b), respectively.

### B. Monte Carlo simulation of breast patient

**Figure 2:**
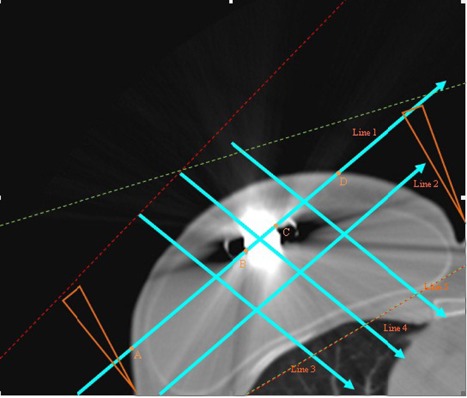
Central CT slice of the breast patient with the tissue expander (including RT isocenter) that was used for the MC simulation. Lines 1–5 represent the axes used herein for the dose profile calculations.

Figure 2 shows the central CT slice of the breast patient with the tissue expander that was used for the simulation runs, and includes the isocenter voxel used for the RT treatment. The lines drawn on this CT slice represent the axes along which the dose profiles were calculated for the two geometries. Line 1 is parallel to the central axis of the two opposed radiation fields and intersects the center of the tissue expander. Line 2 is parallel to line 1, shifted 2.5 cm towards the patient's chest wall. Lines 3, 4 and 5 are perpendicular to Line 1 and placed in 3 cm intervals, as shown in Fig. 2, with line 4 intersecting the center of the expander. A and D are the points where line 1 enters and exits the breast, while B and C are the points where it enters and exits the expander. Line 4 enters the breast at point I, while it intersects the expander's borders at points II and III.

**Figure 3:**
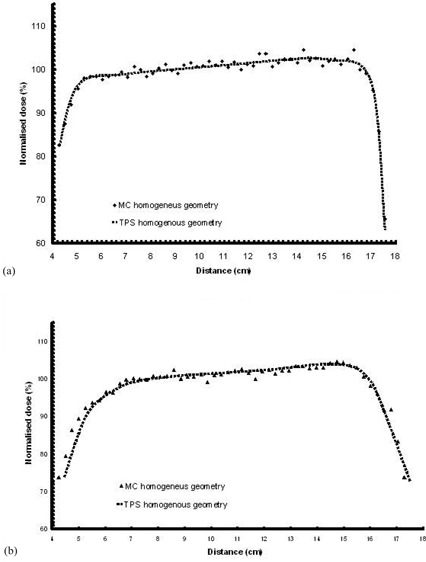
Dose profile along line 1 for: (a) the 6 MV (upper) and (b) the 18 MV modality of the linac (lower). TPS and MC calculations are performed in a homogeneous CT‐based geometry. The dose is normalized to the isocenter of the RT plan. Distance is presented in a “left to right” direction. (See also Fig. 2.)

Figure 3 shows the comparison between the dose profiles for (a) the 6 MV and (b) the 18 MV modes along line 1, calculated by the TPS and the MC simulation for the homogeneous CT‐based geometry. The dose is normalized to the radiation isocenter according to the RT plan, while x‐axis corresponds to the distance along line 1 in a “left to right” direction. Comparison of above profiles reveals a very close agreement between the TPS and the MC results, with differences lying below 3%. This also verifies that the MC simulation is properly attuned to the linac's characteristics. It is noted that the TPS calculations didn't take into consideration the presence of the expander, and that the whole region of the breast was assumed to be uniform with normal breast tissue.

**Figure 4:**
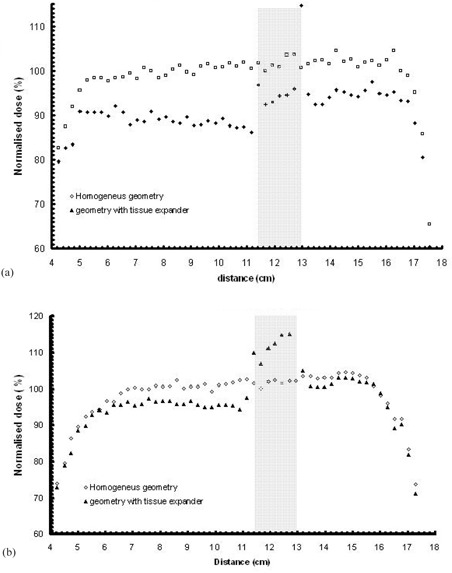
MC calculated dose profile along line 1 for: (a) the 6 MV (upper) and (b) the 18 MV modality of the linac (lower), calculated for the homogenous reconstructed geometry and the CT based geometry including the tissue expander. The grey area outlines the boundaries of the tissue expander. Distance in cm is presented in a “left to right” direction, according to the CT slices. (See also Fig. 2.)

MC results in the homogenous reconstructed breast are used in the following discussion, as the basis to compare with MC results for the reconstructed breast including inhomogeneities and the metallic tissue expander.

(Figure 4a) presents the MC‐calculated dose profiles along line 1 in the two geometries (homogeneous and including inhomogeneities) for the 6 MV mode. The grey area in the graphs outlines the projection of expander material along line 1.

**Figure 5:**
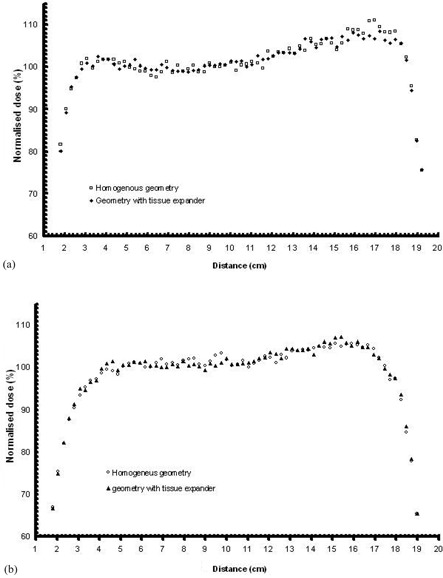
MC calculated dose profile along line 2 for: (a) the 6 MV (upper) and (b) the 18 MV modality of the linac (lower), calcula0ted for the homogenous reconstructed geometry and the CT based geometry including the tissue expander. Distance in cm is presented in a “left to right” direction, according to the CT slices. (See also Fig. 2.)

The presence of the metallic port results in a dose decrease along line 1, in the order of 7%–13%. It is noted that the dose decrease is not only observed at the vicinity of the metallic port, but throughout all the area in the shadow of the metallic port (relative to each photon beam). (Figure 4b) shows corresponding results for the 18 MV modality. A similar but much smoother effect compared with the 6 MV photon beams is observed, as expected, due to the higher photon energies. Dose reduction along line 1 owing to the presence of the tissue expander is not higher than 6% for the 18 MV beams. These results agree well with corresponding experimental measurements by Thompson and Morgan^(4)^ who estimated an underdosage of the order of 10% in a clinical situation using a tangential pair of parallel 6 MV opposed beams, although the underdosage of regions surrounding the magnet was up to ~ 30% for a single 6MV photon beam. This may be compared to results presented by Damast et al.^(2)^ who observed a dose reduction of as much as 22% for a single 6 MV photon beam, and 16% for a 15 MV beam due to the presence of the metallic port.

(Figures 5a)and (5b) present the dose profiles for the 6 MV and 18 MV treatment plans along line 2 (see Fig. 2). Distance on the figures travels “Left to Right”. It can be seen that along this line, where direct photon beam propagation does not intersect with a high‐Z material, dose distribution is practically not affected. Attenuation of primary photon beam remains unchanged, while the contribution of lateral scatter in the absorbed dose along this axis remains also unaffected. Moreover, secondary electrons emerging from the magnetic port have sub‐cm range in water (or saline solution) and are thus absorbed within very short distances, without contributing to dose along line 2.

**Figure 6:**
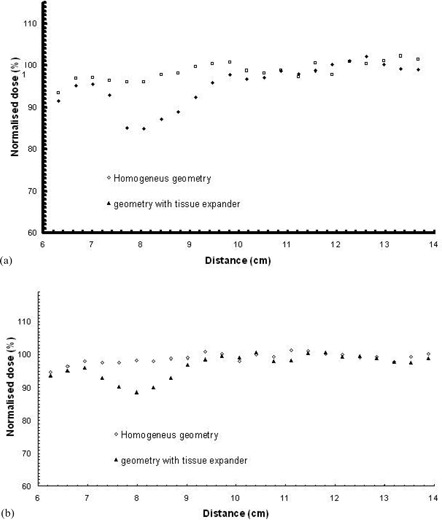
MC calculated dose profile along line 3 for: (a) the 6 MV (upper) and (b) the 18 MV modality of the linac (lower), calculated for the homogenous reconstructed geometry and the CT based geometry including the tissue expander. Distance in cm is presented in an “anterior to posterior” direction, according to the CT slices. (See also Fig. 2.)

**Figure 7:**
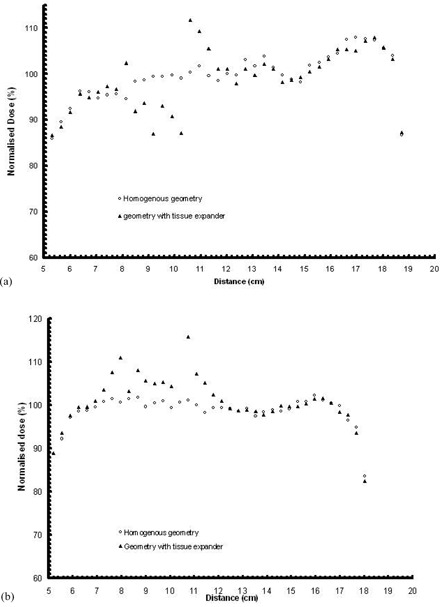
MC calculated dose profile along line 5 for: (a) the 6 MV (upper) and (b) the 18 MV modality of the linac (lower), calculated for the homogenous reconstructed geometry and the CT based geometry including the tissue expander. Distance in cm is presented in a “left to right” direction, according to the CT slices. (See also Fig. 2.)

Dose profiles along line 3 (see Fig. 2) are presented in (Figs. 6a)and (6b), with distance increasing in an “anterior to posterior” direction. A dose reduction is observed along line 2 when it intersects with the area that lies in the shadow of the metallic port (respective to each photon beam direction). Maximum underdosage is observed near the intersection of lines 2 and 3, and is about 11% for the 6 MV and 9% for the 18 MV mode. A similar effect is observed on the dose profiles along line 5, which are presented in (Figs. 7a)and (7b).

**Figure 8:**
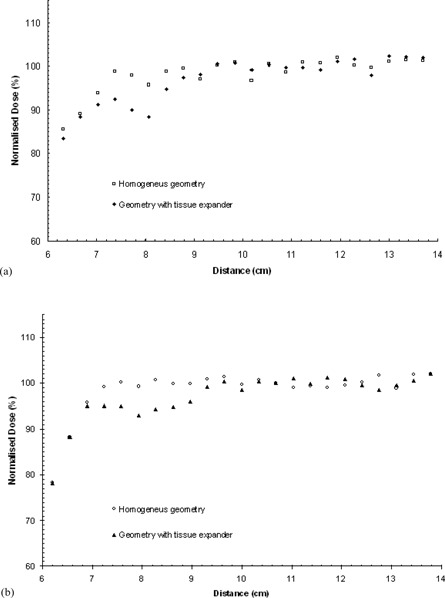
MC calculated dose profile along line 4 for: (a) the 6 MV (upper) and (b) the 18 MV modality of the linac (lower), calculated for the homogenous reconstructed geometry and the CT based geometry including the tissue expander. The grey area outlines the boundaries of the tissue expander. Distance in cm is presented in am “anterior to posterior” direction, according to the CT slices. (See also Fig. 2)

Figure 8 presents the dose profile for line 4 for both modalities of the linac. Line 4 is perpendicular to line 1 and passes through the central region of the metallic port (see Fig. 2). Distance on the figures corresponds to an “anterior to posterior” direction. Similar to line 2, no dose reduction is observed for points that primary photon beams reach without intersecting a high‐Z material. However, MC results show increased doses in the close vicinity of the metallic port. This is mainly attributed to the increased photoelectric effect near the surface of the metallic port, and the high importance of pair production, with emerging secondary electrons depositing their energy within a few mm away from the surface of the magnetic port (for the ${\text{Nd}}_{2}{\text{Fe}}_{14}B$ magnet, pair production becomes the main interaction process for photon energies above 8 MeV). Dose enhancement for the 6 MV beams reaches up to 9% at 2 mm away from the magnet surface, and vanishes at corresponding distances greater than 1 cm (Fig. 8(a). The effect is more pronounced (up to 12%) for the 18 MV photon beams (Fig. 8(b) mainly due to the increased contribution of pair production.

Similar dose perturbation effects have been reported recently by Gossman et al.^(17)^ investigating how vascular ports containing titanium metal alloy affect the delivered dose in external beam RT. Effects of backscatter, lateral scatter and attenuation up to 5.0%, 3.4% and 16.8% for 6 MV, and 7.0%, 7.7% and 7.2% for 18 MV, were respectively observed.

## IV. CONCLUSIONS

The Monte Carlo simulation on a CT‐based geometry of the radiotherapy treatment of a breast patient with the Magna‐Site tissue expander in place was used in order to calculate the dose perturbation on the planned dose distribution caused by the presence of the metallic port. Results show an underdosage of the area lying in the shadow of the metallic port (relative to each beam), which can range between 6%–13% for an RT plan including two tangential 6 MV photon beams (and less pronounced when 18 MV beams are used). Our results agree well with corresponding experimental measurements by Thomson and Morgan,^(4)^ and are relevant to results presented by Damast et al.^(2)^ for a single 6 MV photon beam in a water phantom. Moreover, dose “hot spots” are also observed in the close vicinity of the metallic port (especially for the 18 MV photon beams). This effect should be taken under consideration if the magnetic port is placed close to the expander surface (less than 1 cm away), since unwanted “hot spots” could be created not only in the saline solution, but also in the patient's tissue.

## ACKNOWLEDGEMENTS

This work was supported by a Grant from the Greek National Central Council of Health.
